# Bacillibactin, a Potential *Bacillus*-Based Antibacterial Non-Ribosomal Peptide: In Silico Studies for Targeting Common Fish Pathogens

**DOI:** 10.3390/ijms26125811

**Published:** 2025-06-17

**Authors:** Evgeniya Prazdnova, Anna Zaikina, Alexey Neurov, Maria Mazanko, Anuj Ranjan, Dmitry Rudoy

**Affiliations:** 1 Research Laboratory «Agrobiotechnology Center», Don State Technical University, 344002 Rostov-on-Don, Russiammazanko@sfedu.ru (M.M.);; 2 School of Life Sciences, ITMO University, 197101 Saint Petersburg, Russia; zaikina@scamt-itmo.ru; 3 Amity Institute of Environmental Toxicology Safety and Management, Amity University, Sector 125, Noida 201303, Uttar Pradesh, India; aranjan@amity.edu

**Keywords:** aquaculture probiotics, bacillibactin, fengycin, surfactin, molecular docking, molecular dynamics

## Abstract

Aquaculture is one of the fastest-growing sectors in food production. The widespread use of antibiotics in fish farming has been identified as a driver for the development of antibiotic resistance. One of the promising approaches to solving this problem is the use of probiotics. There are many promising aquaculture probiotics in the *Bacillus* genus, which produces non-ribosomal peptides (NRPs). NRPs are known as antimicrobial agents, although evidence is gradually accumulating that they may have other effects, especially at lower (subinhibitory) concentrations. The mechanisms of action of many NRPs remain unexplored, and molecular docking and molecular dynamics studies are invaluable tools for studying such mechanisms. The purpose of this study was to investigate the in silico inhibition of crucial bacterial targets by NRPs. Molecular docking analyses were conducted to assess the binding affinities of the NRPs of *Bacillus* for protein targets. Among the complexes evaluated, bacillibactin with glutamine synthetase, dihydrofolate reductase, and proaerolysin exhibited the lowest docking scores. Consequently, these complexes were selected for further investigation through molecular dynamics simulations. As a result, three additional potential mechanisms of action for bacillibactin were identified through in silico analyses, including the inhibition of glutamine synthetase, dihydrofolate reductase, and proaerolysin, which are critical bacterial enzymes and considered as the potential antibacterial targets. These findings were further supported by in vitro antagonism assays using bacillibactin-producing *Bacillus velezensis* strains MT55 and MT155, which demonstrated strong inhibitory activity against *Pseudomonas aeruginosa* and *Aeromonas veronii*.

## 1. Introduction

The use of *Bacillus*-based biopreparations is considered a more environmentally friendly alternative to antibiotics, with non-ribosomal peptides (NRPs) being one of the key factors underlying this advantage [1,2]. Although *Bacillus* species are not widespread as human probiotics, they are frequently used in animal husbandry and, in particular, aquaculture [3,4]. *Bacillus* probiotics are capable of producing NRPs and are able to inhibit a broad range of aquaculture pathogens, such as *Pseudomonas* and *Aeromonas*, or enhance host immunity, which makes them promising tools for disease control in fish farming [1].

However, the specific mechanisms of action of some NRPs remain not totally explored [5]. A deeper understanding of these mechanisms could significantly advance the development of aquaculture as a high-tech industry [6,7].

Non-ribosomal peptides (NRPs) are a diverse group of bioactive secondary metabolites produced by various bacterial species [8,9]. They provide the antagonism against pathogenic microorganisms in aquaculture and agriculture [10]. These cyclic lipopeptides have broad-spectrum antimicrobial, antiviral, and antifungal properties, as well as stability and low toxicity [5,11].

In this study, we investigated the action of three NRPs produced by *Bacillus* species—bacillibactin, surfactin, and fengycin. They were chosen because of their abundance, structural diversity, well-characterized activity, such as antagonism to fish pathogens, and their widespread occurrence in *Bacillus* strains used as probiotics in aquaculture [1,9,12]. Two strains capable of producing these metabolites were used in subsequent in vitro studies.

Surfactin is a cyclic heptapeptide linked to a β-hydroxy fatty acid, which is known for its strong antibacterial and antiviral activities [9,12]. Fengycin, a cyclic decapeptide with a β-hydroxy fatty acid chain, has antibacterial and antifungal activity and potential anticancer effects [9,13]. Bacillibactin, a catecholate siderophore that helps cells absorb iron, also has direct antibacterial and antifungal activity [14].

To explore the potential mechanisms of action of these NRPs, we focused on their interactions with several essential bacterial proteins from common bacterial fish pathogens. These proteins are involved in cell wall biosynthesis, metabolism, and virulence.

The pathogenic species *Pseudomonas aeruginosa* and *Aeromonas veronii*, which are the most common in fish, are often resistant to antibiotics and can reduce aquaculture productivity. Therefore, they were selected for in vitro testing as model pathogens [15,16].

The following protein targets were selected in *Aeromonas hydrophila*: proaerolysin, D-alanine–D-alanine ligase, and S-adenosylmethionine synthase. Proaerolysin is a precursor of the pore-forming toxin from *Aeromonas hydrophila*, which disrupts target cell membranes and thereby ensures virulence [17]. D-alanine–D-alanine ligase catalyzes the ATP-dependent formation of the D-alanyl–D-alanine dipeptide, a key step in peptidoglycan biosynthesis [18]. S-adenosylmethionine synthase (methionine adenosyltransferase) generates S-adenosylmethionine. It is the main methyl donor in cellular metabolism, influencing gene expression and cell proliferation [19].

For *Staphylococcus xylosus*, glutamine synthetase was selected, which catalyzes the ATP-dependent biosynthesis of glutamine from glutamate and ammonia. It plays a central role in nitrogen metabolism and transcriptional regulation [20].

Dihydrofolate reductase, a key enzyme in folate metabolism, was selected for *Vibrio anguillarum*. This enzyme is involved in the de novo synthesis of glycine and purines, which are required for nucleotide biosynthesis and cell growth [21].

The following targets were selected for *Streptococcus agalactiae*: phosphopentomutase, lipoprotein signaling peptidase, catabolism control protein A, and ribosome large subunit protein L19. Phosphopentomutase takes part in nucleotide metabolism and the pentose phosphate pathway, interconverting ribose 5-phosphate and ribose 1-phosphate [22]. Lipoprotein signal peptidase specifically catalyzes the removal of signal peptides from prolipoproteins [23]. Catabolism control protein A is a global transcriptional regulator of carbon catabolite repression and carbon catabolite activation that ensures optimal energy utilization under different conditions [24]. The ribosome large subunit protein L19 is located at the junction of the 30 S–50 S ribosomal subunits and plays a role in the structure and function of the aminoacyl-tRNA binding site [25].

The aim of this research was to clarify the mechanisms of *Bacillus* metabolites’ action against fish pathogens. An in silico approach was used to identify the potential additional activities of NRPs, and further validation of the binding of these proteins by molecular dynamics (MD) simulation studies was performed. In vitro antagonism tests were also performed using NRP-producing *Bacillus velezensis* strains.

## 2. Results

### 2.1. Docking Studies

**Figure 1:**
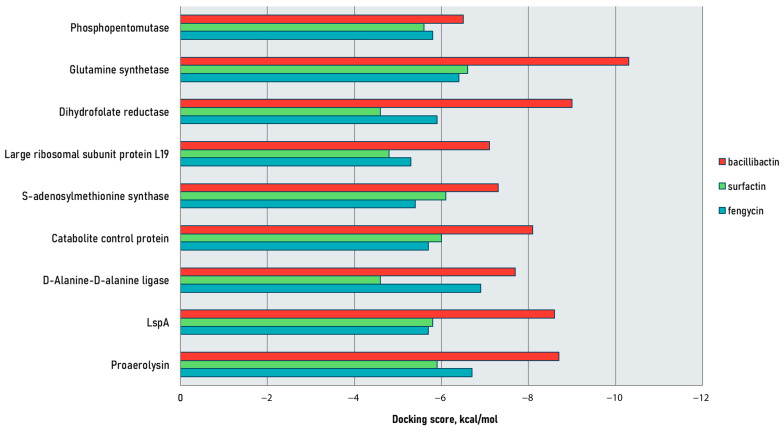
Binding energies (in Kcal/mol) of selected non-ribosomal peptides against target proteins calculated by molecular docking using Autodock Vina.

The docking scores obtained after molecular docking of the selected NRPs are summarized in Figure 1 and Table 1. Geometric constraints, such as distance and atomic orientation, were also considered when identifying productive poses [26,27]. See Tables S1–S3 for detailed descriptions of productive poses; green indicates d ≤ 3.0 Å and θ ≥ 120°, and yellow stands for d = 3.1–3.4 Å and θ = 90–119°. Table S4 provides grid-box dimensions and axes.

Bacillibactin exhibited the highest binding affinity across all proteins, with the lowest dock score of −10.3 kcal/mol for glutamine synthetase. Fengycin and surfactin had relatively lower binding affinities, with dock scores ranging from −4.6 to −6.9 kcal/mol. Among the protein targets, fengycin had the weakest binding to the large ribosomal subunit protein L19, with a dock score of −5.3 kcal/mol, while surfactin had the weakest binding to dihydrofolate reductase and D-alanine–D-alanine ligase, with a dock score of −4.6 kcal/mol. These results suggest that bacillibactin may be the most potent inhibitor of the given proteins among the tested ligands.

The results also demonstrate that bacillbactin, fengycin and surfactin interact with key binding site residues of glutamine synthetase (Lys 201, Arg 323, and His 248), proaerolysin (Asp 360, Pro 347, and Arg 356), and dihydrofolate reductase (Leu 86, Gly 88, Gly 38, His 39, Arg 59, and Pro 87). It is illustrated in Tables S1–S3, and residues within or near the catalytic site are marked in red.

#### 2.1.1. Bacillibactin

**Figure 2:**
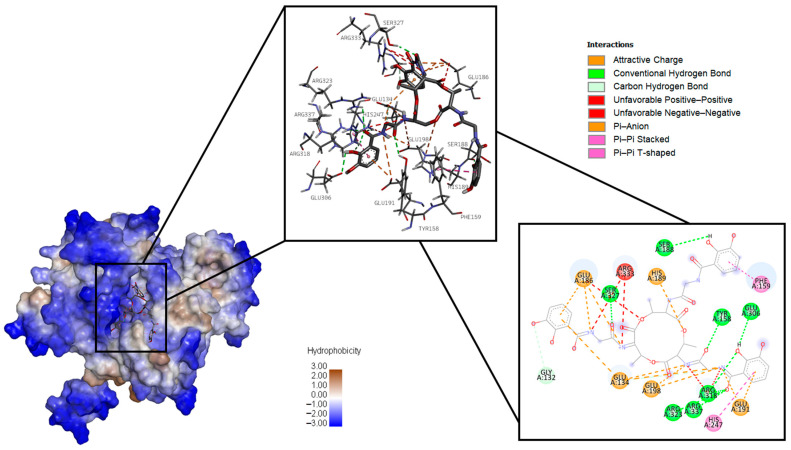
Three-dimensional diagram of the location of bacillibactin in the binding site of glutamine synthetase on the left, three-dimensional diagram of glutamine synthetase amino acid interaction with bacillibactin in the middle, and two-dimensional diagram of glutamine synthetase amino acid interaction with bacillibactin on the right.

**Figure 3:**
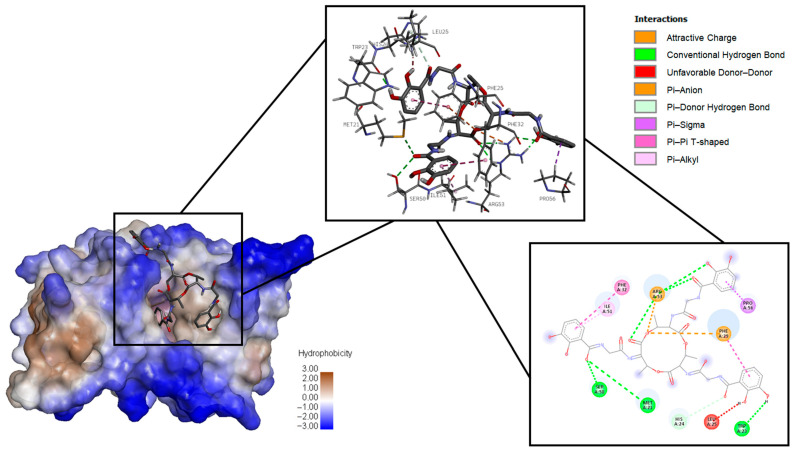
Three-dimensional diagram of the location of bacillibactin in the binding site of dihydrofolate reductase on the left, three-dimensional diagram of dihydrofolate reductase amino acid interaction with bacillibactin in the middle, and two-dimensional diagram of dihydrofolate reductase amino acid interaction with bacillibactin on the right.

**Figure 4:**
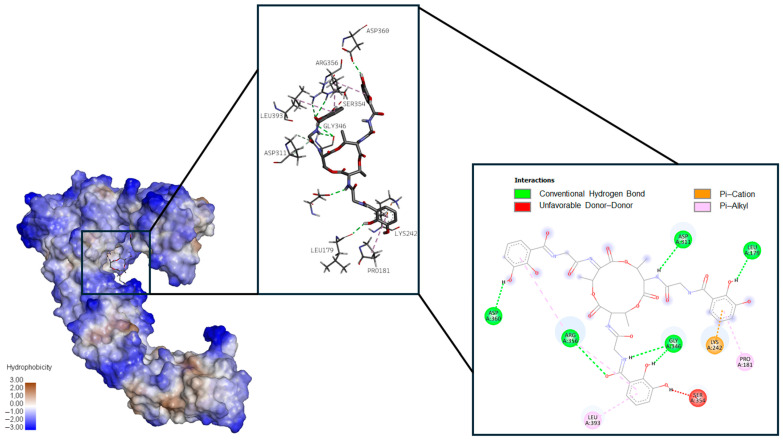
Three-dimensional diagram of the location of bacillibactin in the binding site of proaerolysin on the left, three-dimensional diagram of proaerolysin amino acid interaction with bacillibactin in the middle, and two-dimensional diagram of proaerolysin amino acid interaction with bacillibactin on the right.

Figure 2, Figure 3 and Figure 4, respectively, present 3D and 2D diagrams of the complexes with bacillibactin.

The following types of bonds were formed after the docking of surfactin and glutamine synthetase: conventional hydrogen bonds with Tyr 158, Arg 318, Arg 323, Ser 327, Arg 337, Ser 188, and Glu 306; carbon–hydrogen bonds with Gly 132 and Glu 134; attractive charge with His 189, Glu 186, Glu 134, Glu 198, and Glu 191; unfavorable positive–positive with Arg 318 and Arg 333; and unfavorable negative–negative with Glu 186. Some Pi bonds also were formed: Pi–anion (Glu 134, Glu 186, Glu 191), Pi–Pi stacked (Phe 159), and Pi–Pi T-shaped (His 247).

Bacillibactin formed conventional hydrogen bonds (Arg 53, Met 21, Ser 50, Trp 23) and attractive charge (Arg 53), unfavorable donor–donor (Leu 25), Pi–anion (Phe 29), Pi–donor (His 24), Pi–sigma (Pro 56), Pi–Pi T-shaped (Phe 29, Phe 32), and Pi–alkyl (Ile 51) bonds with dihydrofolate reductase.

Bacillibactin formed conventional hydrogen bonds (Asp 311, Leu 179, Gly 346, Arg 356, Asp 360), unfavorable donor–donor (Ser 354) bonds, and two types of Pi–bonds, i.e., Pi–cation (Lys 242) and Pi–alkyl (Pro 181, Leu 393), with proaerolysin.

#### 2.1.2. Fengycin

**Figure 5:**
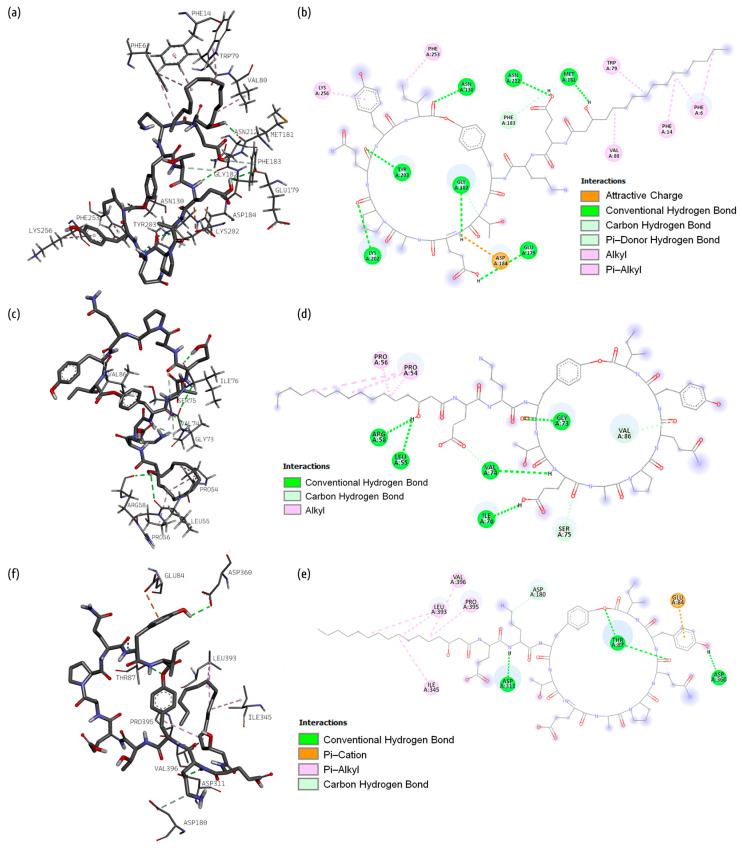
Three-dimensional diagrams of fengycin docked in the binding site of (**a**) glutamine synthetase, (**c**) dihydrofolate reductase, and (**e**) proaerolysin. Two-dimensional plots of the interaction of (**b**) glutamine synthetase, (**d**) dihydrofolate reductase, and (**f**) proaerolysin amino acids with fengycin.

Figure 5 presents 3D and 2D diagrams of the complexes with fengycin.

The following interactions between glutamine synthetase and fengycin were observed during the molecular docking process: conventional hydrogen bonds were contributed by Gly 182, Glu 179, Met 181, Asn 130, Lys 202, Tyr 203, and Asn 212, while a carbon–hydrogen bond was contributed by Gly 182, an alkyl bond was contributed by Val 80, an attractive charge was contributed by Asp 184, a Pi–donor bond was contributed by Phe 183, and Pi–alkyl bonds were contributed by Lys 256, Phe 6, Phe 14, Trp 79, and Phe 253.

Fengycin formed four conventional hydrogen bonds with Val 74, Ile 76, Leu 55, Arg 58, and Gly 73, four carbon–hydrogen bonds with Gly 73, Val 74, Ser 75, and Val 86, and alkyl bonds with Pro 54 and Pro 56 of dihydrofolate reductase.

Fengycin formed three conventional hydrogen bonds with Asp 311, Thr 87, and Asp 360, one carbon–hydrogen bond with Asp 180, and one Pi–anion bond with Glu 84 of proaerolysin. Some alkyl bonds were also formed (Leu 393, Val 396, Pro 395, Ile 345).

#### 2.1.3. Surfactin

**Figure 6:**
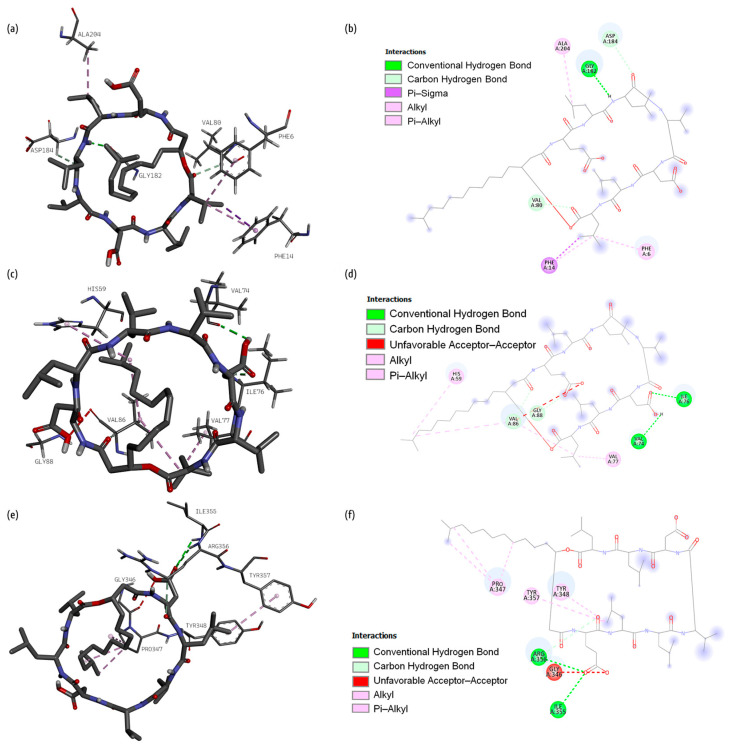
Three-dimensional diagrams of the surfactin docked in the binding site of (**a**) glutamine synthetase, (**c**) dihydrofolate reductase, and (**e**) proaerolysin. Two-dimensional plots of the interaction of (**b**) glutamine synthetase, (**d**) dihydrofolate reductase, (**f**) proaerolysin amino acids with surfactin.

Figure 6 presents 3D and 2D diagrams of the complexes with surfactin.

The following types of bonds were formed after the docking of surfactin and glutamine synthetase: conventional hydrogen bonds (Gly 182), carbon–hydrogen bonds (Val 80, Asp 184), and Pi–sigma (Phe 14), alkyl (Ala 204), and Pi–alkyl bonds (Phe 6, Phe 14).

Surfactin formed conventional (Val 74, Ile 76) and carbon (Val 86, Gly 88) hydrogen bonds, alkyl bonds (Val 86, Val 77), unfavorable acceptor–acceptor (Val 86), and Pi–alkyl bond (His 59) with dihydrofolate reductase.

It also formed conventional (Arg 356, Ile 355) and carbon (Arg 356) hydrogen bonds and Pi–alkyl bonds (Tyr 348, Tyr 357) with proaerolysin. Unfavorable acceptor–acceptor (Gly 346) and alkyl (Pro 347) bonds were formed as well.

### 2.2. Molecular Dynamics Simulation

**Figure 7:**
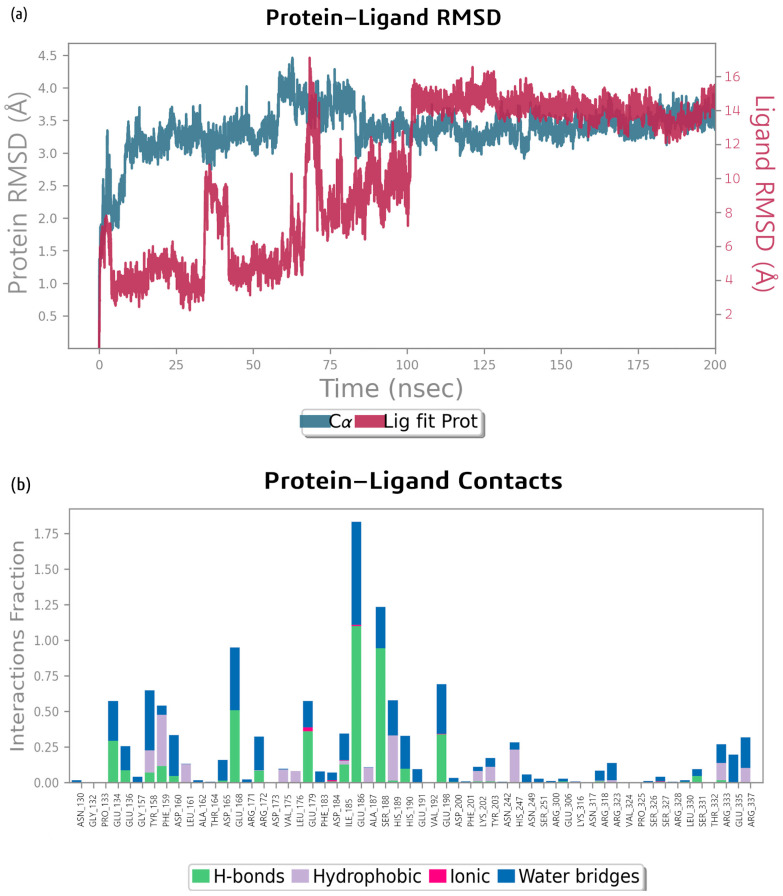

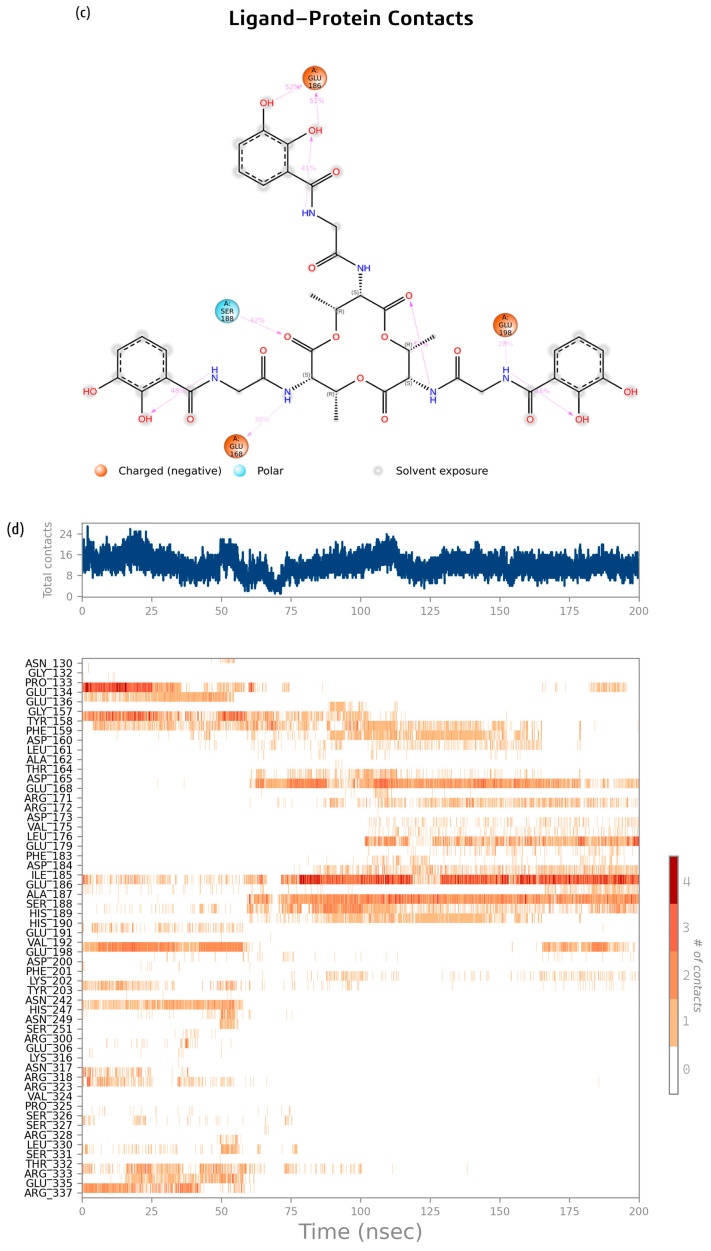
(**a**) RMSD analysis of the molecular dynamics (MD) simulation trajectories generated using Desmond for bacillibactin and glutamine synthetase, (**b**) analysis of types of contacts between bacillibactin and glutamine synthetase, (**c**) protein–ligand contact plots for the glutamine synthetase–bacillibactin complex, and (**d**) protein–ligand contact plots throughout the MD simulation of bacillibactin with glutamine synthetase.

The molecular dynamics simulation of the bacillibactin–glutamine synthetase complex performed using Desmond 3.5 revealed that the root mean square deviation (RMSD) of Cα atoms (asymmetric carbon atoms to which various substituents in the amino acid are attached) of the glutamine synthetase protein backbone relative to its initial position increased dramatically to 3.3 Å during the first 10 ns, increased again at 60 ns to 4 Å after a period of stability, and stabilized almost completely at 80 ns. The RMSD of the ligand increased and decreased sharply at approximately 40 ns and 70 ns. This indicates that the position of the ligand changed, but it appeared to be unstable. The RMSD started to increase gradually at 75 ns and finally stabilized at 100 ns (Figure 7a).

The MD simulation also provided the data on molecular interaction and type of contacts, which are presented in Figure 7b, where the interaction fractions between bacillibactin and various residues of glutamine synthetase are presented. Amino acid residues, such as Glu 186, Ser 188, Glu 168, Glu 134, and Glu 198, contribute most to the formation of H-bonds in the bacillibactin–glutamine synthetase complex. Most of the amino acid residues form water bridges. A portion of the protein is involved in hydrophobic interactions, especially Phe 159, His 189, and His 247. Glu 179 is involved in ionic interactions. Figure 7c shows the residue interactions of glutamine synthetase with bacillibactin. Interactions lasting more than 25% of the simulation time were considered. Ser 188 formed a polar bond for 42% of the simulation. Glu 186 formed two negative interactions for 52% and 51% of the simulation. Glu 198 (28%) and Glu 168 (30%) also formed charged negative interactions with bacillibactin. Glu 49 formed two negative bonds with bacillibactin, which remained for 49% and 47% of the simulation time. A Glu 72 negative bond occurred for 45% of the simulation time. The average number of bonds between bacillibactin and glutamine synthetase throughout the simulation was approximately 16. The diagram (Figure 7d) shows the dynamic formation of bonds. Before the stabilization of the complex, bonds with Glu 134, Glu 136, Phe 159, Tyr 158, Glu 198, His 247, and Arg 337 were preserved. After approximately 75 ns, the complex was stabilized by other bonds: Asp 16, Glu 168, Glu 179, Glu 186, Ser 188, His 189, and His 190.

**Figure 8:**
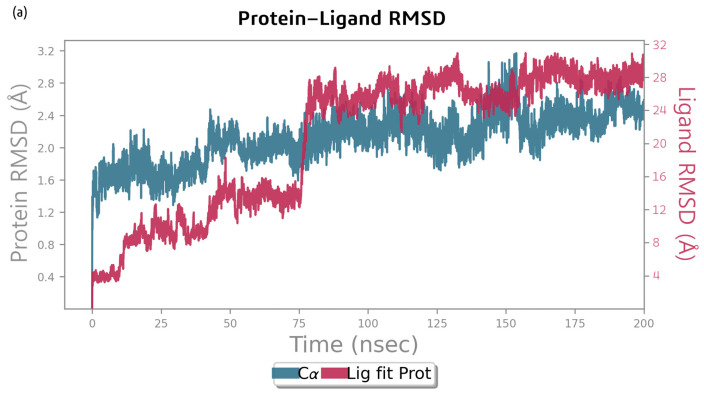

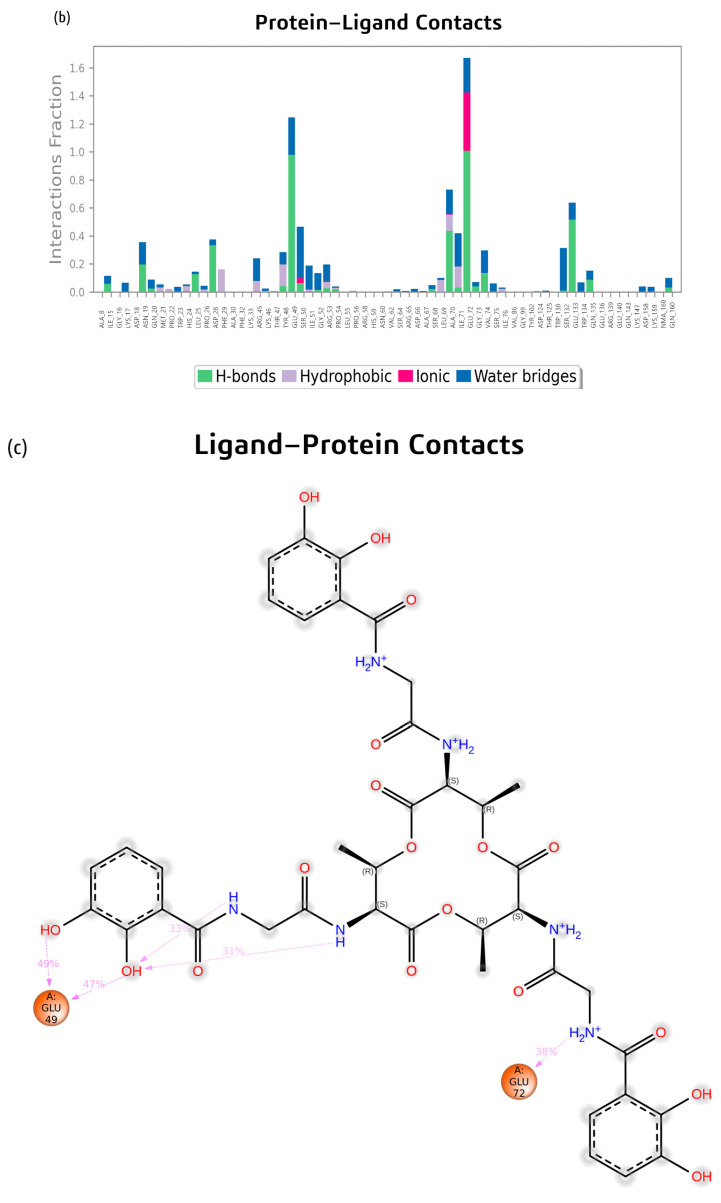

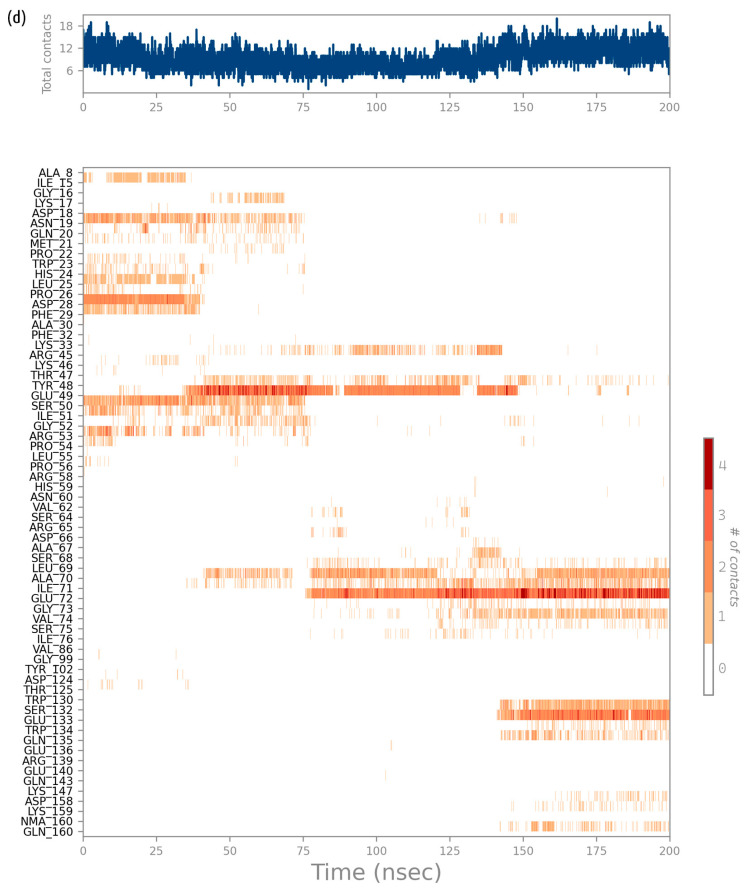
(**a**) RMSD analysis of the MD simulation trajectories generated using Desmond for bacillibactin and dihydrofolate reductase, (**b**) analysis of types of contacts between bacillibactin and dihydrofolate reductase, (**c**) protein–ligand contact plots for the dihydrofolate reductase–bacillibactin complex, and (**d**) protein–ligand contact plots throughout the MD simulation of bacillibactin with dihydrofolate reductase.

The RMSD plot for the MD simulation of the bacillibactin–dihydrofolate reductase complex in Figure 8 shows that the protein underwent significant conformational changes, with its RMSD increasing from approximately 1.0 Å to 2.5–3.0 Å over 200 ns. The ligand maintained a relatively stable interaction with the protein, with its RMSD stabilizing at approximately 1.5–2.5 Å. The RMSD of the ligand increased at the start of the simulation to 0.4 Å, increased again to 0.8 Å after 12 ns, and increased by 45 ns to 1.3 Å. At 75 ns, the position abruptly changed to 2.8 Å. Both the protein and ligand RMSD values fluctuated but stabilized after approximately 100–150 ns, indicating that the system reaches equilibrium. The RMSD of the protein backbone relative to its starting position increased abruptly to 1.6 Å, gradually increased to approximately 2.5 Å throughout the simulation, and remained relatively stable afterwards until 200 ns.

The MD simulation of the bacillibactin–dihydrofolate reductase complex revealed that amino acid residues, such as Glu 49, Glu 72, Ala 70, Glu 133, Asp 28, and Asn 19, contribute most to H-bond formation. Glu 72 also forms ionic bonds and water bridges. The interactions are shown in Figure 8b. Figure 8c shows the residue interactions of dihydrofolate reductase with bacillibactin. Interactions lasting more than 30% of the simulation time were considered. Glu 49 formed two negative bonds with bacillibactin, which remained for 49% and 47% of the simulation time. A Glu 72 negative bond occurred for 45% of the simulation time. On average, 12 bonds were formed between bacillibactin and dihydrofolate reductase. After 75 ns, bonds with Glu 72 were formed. After 140 ns, the Glu 133 and Ser 132 bonds were clearly visible, but the bonds with Glu 49 disappeared. Overall, there were fewer stable interactions than the complex of glutamic synthetase and bacillibactin. The interactions are shown in Figure 8d.

**Figure 9:**
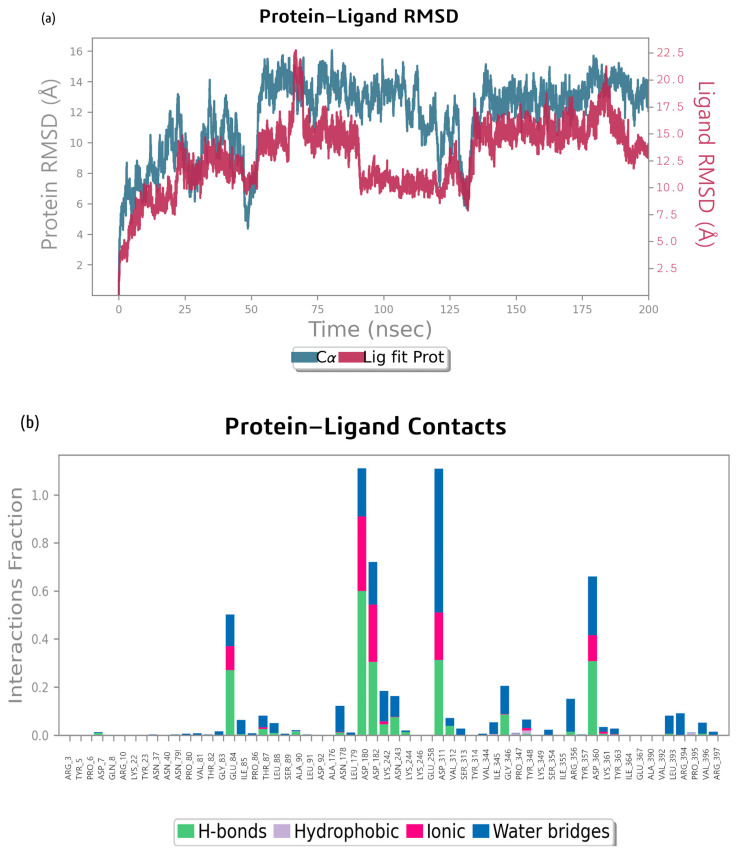

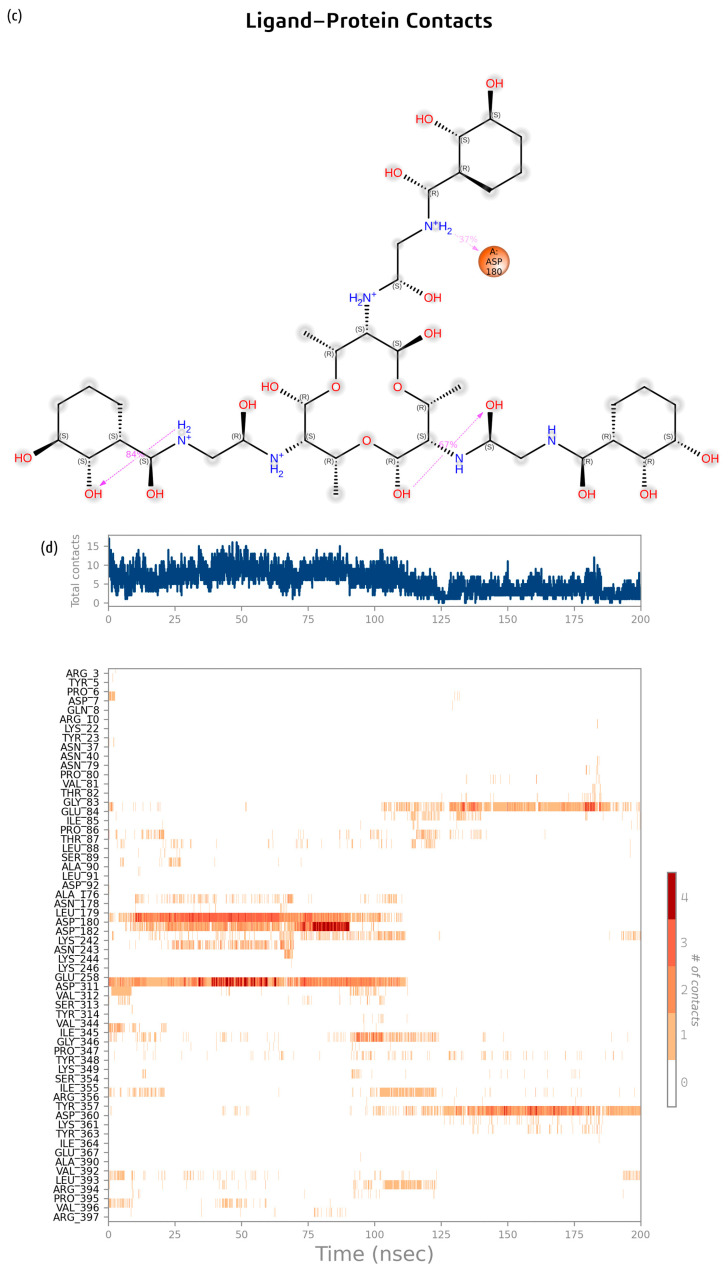
(**a**) RMSD analysis of the MD simulation trajectories generated using Desmond for bacillibactin and proaerolysin, (**b**) analysis of types of contacts between bacillibactin and proaerolysin, (**c**) protein–ligand contact plots for the proaerolysin–bacillibactin complex, and (**d**) protein–ligand contact plots throughout the MD simulation of bacillibactin with proaerolysin.

The RMSD plot for the bacillibactin–proaerolysin complex shows that the protein’s RMSD increased from approximately 2.0 Å to approximately 9.0 Å over 200 ns, indicating significant conformational changes. The RMSD of proaerolysin was relatively constant from 50 ns to 110 ns. After a decrease, at 140 ns, the position of the protein stabilized. The RMSD for the ligand increased smoothly to 10 Å after 60 ns and then increased and decreased sharply. From 90 ns to 130 ns, the RMSD was kept at 7.5 Å, and from 130 ns to 175 ns, it was again set at 10 Å. At the end of the simulation, the ligand position destabilized slightly (Figure 9a).

As shown in Figure 9b, the amino acids Glu 84, Leu 179, Asp 180, Asp 311, and Tyr 357 play a significant role in the formation of H- and ionic bonds along with several water bridges in the proaerolysin–bacillibactin complex. Figure 9c shows the residue interactions of proaerolysin with bacillibactin. Interactions lasting more than 30% of the simulation time were considered. The key residue involved in the formation of the proaerolysin–bacillibactin complex is Asp 180. Asp 180 formed a negative interaction with bacillibactin, which remained for 37% of the simulation time. In the bacillibactin–proaerolysin complex, up to 125 ns, the total number of bonds was approximately 4, and afterwards, it decreases to 2. In the first half of the simulation, the largest number of bonds was formed with Leu 179, Asp 180, and Asp 311. After 125 ns, the old bonds were broken, and new bonds were formed with Gly 83 and Asp 360. There were fewer stable bonds than in the first two complexes. The interactions are shown in Figure 9d.

### 2.3. Bacillibactin Genes in Bacterial Genomes

Functional analysis of the two sequenced probiotics’ genomes revealed that they had bacillibactin synthesis genes in their genome. Surfactin and fengycin were also among their potential metabolites. Antismash analysis showed 100% similarity for bacillibactin, bacilysin, macrolactin H, bacillaene, and fengycin and 91% similarity for surfactin and plantazolicin in both the MT55 and MT155 genomes.

### 2.4. In Vitro Exneriments

It was determined that strains with bacillibactin genes (MT55, MT155) had significant antimicrobial activity against *P. aeruginosa* and *A. veronii*. The data on their activity are provided in Table 2.

Thus, strains possessing the bacillibactin gene showed antimicrobial activity against *P. aeruginosa* and *A. veronii*. Both strains demonstrated a high degree of activity against *P. aeruginosa* (100% inhibition) and, to a lesser extent, against *A. veronii*.

## 3. Discussion

*Bacillus* species have previously demonstrated significant antagonistic activity against the fish pathogens listed in this work—*A. hydrophila* [28,29], *P. aeruginosa* [29,30], *S. xylosus*, *V. anguillarum*, and *S. agalactiae* [31].

Our study provides evidence that *Bacillus*-derived NRPs, especially bacillibactin, may act through the direct inhibition of essential bacterial enzymes.

According to the results of our molecular docking experiments, bacillibactin showed the strongest binding affinities among the tested NRPs, with docking scores as low as −10.3 kcal/mol for glutamine synthetase, and similarly high affinity for dihydrofolate reductase and proaerolysin. The stability of the best-performing complexes was verified using MD simulations, which confirmed their stability. The results are particularly remarkable for glutamine synthetase, where bacillibactin maintained an average of 16 stable interactions throughout the simulation. The residues Glu 186, Ser 188, and Glu 198 were crucial for hydrogen bonding and ionic interactions. With dihydrofolate reductase, bacillibactin formed an average of 12 stable bonds, with critical residues Glu 49 and Glu 72 playing roles in them. The bacillibactin complex with proaerolysin demonstrated fewer stable interactions, with residues like Asp 180 and Leu 179 critical for binding.

The stability of the complexes with bacillibactin suggests the potential for this NRP to inhibit the enzymes involved in nitrogen metabolism, folate synthesis, and toxin activation, thereby disrupting essential cellular processes in *A. hydrophila*, *S. xylosus*, *V. anguillarum*, and *S. agalactiae*.

Previous studies usually attributed the antimicrobial action of bacillibactin to its siderophore properties and iron sequestration [32]. However, it was found that bacillibactin can inhibit bacterial growth even in iron-rich conditions [2]. This fact, in line with our findings, allows for implicating direct enzyme inhibition as an additional mechanism to iron binding.

Notably, bacillibactin has been predicted to inhibit proteins such as diguanylate cyclase and biofilm-associated proteins in other pathogens, with docking scores in the range of −6.3 to −7.3 kcal/mol [33], which are comparable but less favorable than the values observed in our study for fish pathogen targets. A molecular docking and MD study [34] suggested that bacillibactin can also inhibit superoxide dismutase in *Micrococcus luteus*.

Fengycin and surfactin also formed stable complexes with some targets. Fengycin achieved a docking score of −6.9 kcal/mol with D-alanine–D-alanine ligase, −6.7 kcal/mol with proaerolysin, and −6.4 with glutamin synthetase, suggesting possible inhibitory effects beyond membrane disruption.

Surfactin bonded most strongly with glutamine synthetase (−6.6 kcal/mol). Therefore, fengycin could interact with two vital protein targets in *A. hydrophila* and one target in *S. xylosus*, inhibiting them. Surfactin targets the same protein, glutamine synthetase, in *S. xylosus*.

For fengycin and surfactin, their primary mechanism of action is usually stated as membrane disruption [35,36,37], but recent findings suggest they can act through quorum sensing inhibition and intracellular target modulation [38]. Our data extend these observations by showing that both compounds can form stable complexes with specific enzymes, although with less potency than bacillibactin.

To date, only a few studies have reported direct inhibition of these specific targets in pathogens by natural compounds. Classic inhibitors of D-alanine–D-alanine ligase (e.g., vancomycin) and dihydrofolate reductase (e.g., trimethoprim) exhibit binding energies in a similar range to those observed for bacillibactin (−8–6 kcal/mol), underscoring its potential as a lead compound for novel antimicrobial development [39,40].

Our in vitro experiments confirm that bacillibactin-producing strains showed antimicrobial activity against the common fish pathogens *P. aeruginosa* and *A. veronii*. This activity supports the in silico predictions. Therefore, bacillibactin can be regarded as a marker for potential probiotic selection.

The multi-targeted inhibition observed here may also explain the broad-spectrum activity of *Bacillus* probiotics producing these substances, as reported in numerous in vivo studies, e.g., [41].

Further biochemical validation of enzyme inhibition and in vivo efficacy studies are obviously required to validate these mechanisms and assess their impact on fish health and pathogen resistance in aquaculture systems.

Speculating on future research directions, engineering *Bacillus* strains for enhanced bacillibactin production or combining NRPs with complementary modes of action could further improve disease control in aquaculture. Furthermore, the identification of stable interactions between bacillibactin and critical enzymes opens avenues for the development of postbiotic formulations or synthetic analogs tailored for specific pathogens, not only in aquaculture but also for managing other animal and human diseases. Taking into account the increasing challenge of antibiotic resistance, such strategies could provide alternatives to conventional antimicrobials.

## 4. Materials and Methods

### 4.1. Preparation of Receptors and Ligands

The crystal structure of proaerolysin was extracted from the RCSB protein data bank (https://www.rcsb.org) access date: 10 October 2023, PDB ID:1PRE [42,43], in .pdb format. The structure was imported into Discovery Studio Visualizer 3.0 (*Dassault Systèmes BIOVIA, San Diego, CA, USA: 2017*), and the structure was cleaned by removing heteroatoms. UCSF Chimera [44] was used for energy minimization, using 50 steps of steepest descent and conjugate gradient. Then, the structural analysis and verification server was used to assess the quality of the structure of proaerolysin. The amino acid sequence of S-adenosylmethionine synthase (accession number A0A7G1L3S3) was retrieved from UniProt [45]. Since its crystal structure was not available in the RCSB protein database, the AlphaFold2 [46,47] model was used for further research. Similarly, for the same reason, homology models of dihydrofolate reductase, D-alanine–D-alanine ligase, lipoprotein signal peptidase, catabolite control protein A, large ribosomal subunit protein L19, phosphopentomutase, and glutamine synthetase were prepared by the SWISS-MODEL [48] online program (https://swissmodel.expasy.org/), access date: 2 November 2023. All the structures were checked for missing side chains, loops, and residues. Hydrogen was added, and the structures were stored for further work. Grid preparation was conducted based on the binding site information using the Autodock tool 1.5.7 [49], and files were stored in .pdbqt file format. Charges and polar hydrogens were added during the preparation process. The 3D structures of bacillibactin, fengycin, and surfactin were retrieved from the ChemSpider database in the structure data file format .mol. Ligand preparation was conducted using Open Babel 3.11 [50] to convert all the compounds into .pdbqt files.

### 4.2. Docking Studies

AutoDock Vina 1.2.4 was used to perform molecular docking of the compounds with the protein targets. During molecular docking, putative ligand binding conformations were generated at the active site of the protein. A docking score was used to rank these conformations in terms of the tightness of the resulting complex. This metric allows for evaluating different complexes according to binding free energy and binding affinity [51].

Vina was run through the command line with a configuration of a grid point spacing of 0.375 Å. The exhaustiveness value used was 8. The docked protein-ligand complexes were subjected to Discovery Studio Visualizer to analyze interactions among them, and all the protein-ligand interaction diagrams were plotted in 2D and 3D. The geometric thresholds were as follows: strong H-bond D (H)…A (maximum distance): 3.4, salt bridge D (H)…A (maximum distance): 4, weak H-bond D (H)…A (maximum distance): 3.8; acceptor–acceptor (maximum distance): 3, hydrogen-bond D-H-A (minimum degree): 90, and hydrogen-bond D-H-A (maximum degree): 180.

Binding sites with key residues were determined for glutamine synthetase (Lys 201, Arg 323, and His 248) [52], proaerolysin (Asp 360, Pro 347, and Arg 356) [53], and dihydrofolate reductase (Leu 86, Gly 88, Gly 38, His 39, Arg 59, and Pro 87) [54].

### 4.3. Molecular Dynamics Simulation

Molecular dynamics simulations were performed for the most stable complexes of NRPs and protein targets using Desmond 3.5 software. The simulation time was 200 ns (nanoseconds). The complexes were solvated in a 10 × 10 × 10 Å^3^ orthorhombic box, and TIP3P water molecules were added. The whole system was neutralized by adding counter ions Na^+^ and Cl^−^ to balance the net charge of the system. Overall, the bacillibactin and glutamine synthetase complex included 16,397 water molecules, 63 Na^+^, and 46 Cl^−^; bacillibactin and dihydrofolate reductase included 6584 water molecules, 21 Na^+^, and 18 Cl^−^; and bacillibactin and proaerolysin complex included 24,343 water molecules, 70 Na^+^, and 68 Cl^−^. Overall, the bacillibactin and glutamine synthetase complex included 16,397 water molecules, 63 Na^+^, and 46 Cl^−^; bacillibactin and dihydrofolate reductase complex/system included 6584 water molecules, 21 Na^+^, and 18 Cl^−^; and bacillibactin and proaerolysin complex included 24,343 water molecules, 70 Na^+^, and 68 Cl^−^.

The minimized system was relaxed with a number of atoms and a pressure and temperature ensemble. The full system was composed of 22,418 atoms; the temperature was maintained at 300 K, and the pressure was maintained at 1.01325 bar.

### 4.4. Probiotics Strains for In Vivo Testing

Two strains of probiotic bacteria previously isolated from the bottom sediments of the Don River, Rostov-on-Don, Russia [55], were used. All strains’ genomes were sequenced using Nanopore technology to identify them and evaluate their biosynthetic potential [56]. Strains were identified as *Bacillus velezensis*. Secondary metabolite synthesis genes were analyzed using the Antismash v7 online tool.

### 4.5. Evaluation of Antimicrobial Activity of Strains

Prior to the experiment, overnight cultures of all test strains of fish pathogens (*Pseudomonas aeruginosa*, *Aeromonas veronii*) were prepared on respective liquid nutrient media. Incubation was conducted at 37 °C in a shaking incubator.

Overnight cultures of bacilli were also prepared. For this, 5 mL of liquid LB medium was added to a test tube, a loop of the strain biomass was inoculated, and then it was placed on an incline to increase the surface area of the medium. Incubation was carried out at 37 °C. A biofilm developed at the surface in all samples. After 24 h, the test tubes were centrifuged (5 min, 3900 rpm), and the supernatant was filtered through a cellulose filter with a pore diameter of 0.22 µm to separate the supernatant from the remaining bacilli cells.

In the well of a sterile 96-well plate, 200 µL of sterile supernatant was placed. Then, 100 µL of the sample was taken from it and placed in the next well. In the same well, 100 µL of nutrient medium was added and thoroughly mixed. Subsequently, 100 µL of the two-fold diluted sample was taken from the well again, and the described actions were repeated. This way, a series of wells with consecutive two-fold dilutions was obtained. Wells with nutrient medium without supernatant were used as controls.

The biomass of pathogen strains from the overnight culture was centrifuged (5 min, 3600 rpm) and resuspended in fresh nutrient medium to an OD600 of 0.1. Then, 100 µL of the obtained suspension was added to each well. Each plate was sealed with a sterile plate seal and placed in a thermostat at a temperature of 37 °C for 24 h.

After 24 h, the growth of pathogen strains in the presence of each supernatant concentration was evaluated. The degree of cell growth was assessed based on the optical density at a wavelength of 600 nm using a multimodal plate reader SpectroSTAR Omega (BMG LABTECH, Ortenberg, Germany).

Each experimental variant was carried out in triplicate, and then the standard deviation was calculated.

## 5. Conclusions

Our study demonstrates that bacillibactin can directly inhibit multiple crucial enzymes in aquaculture pathogens, targeting multiple bacterial pathways and providing a molecular basis for its strong antimicrobial activity. Bacillibactin demonstrated the strongest binding affinities among the studied compounds across the target proteins of four common fish pathogens. Fengycin and surfactin also demonstrated potential for inhibiting activity against two and one pathogen, respectively. Although bacillibactin is usually thought to act as a competitive siderophore, our research revealed a potential additional mechanism of its action. Our in vitro experiments confirm that typical bacillibactin-producing strains show antimicrobial activity against common fish pathogens.

The use of *Bacillus*-based probiotics is a promising strategy for controlling common fish pathogens in aquaculture. Delving into the mechanisms of action of NRPs holds significance, as their understanding will allow for the more efficient use of probiotics, the design of constructs for genetic engineering, the more thorough selection of probiotic strains in aquaculture, and the creation of postbiotic formulations.

## Figures and Tables

**Table 1:** Dock scores of the binding energies of protein-ligand determined using AutoDock Vina.

Pathogen	Protein Target	Ligand Dock Score, kcal/mol		
		Bacillibactin	Surfactin	Fengycin
*Aeromonas hydrophila*	Proaerolysin	−8.7 *	−5.9	−6.7 *
	D-alanine–D-alanine ligase	−7.7 *	−4.6	−6.9 *
	S-adenosylmethionine synthase	−7.3 *	−6.1	−5.4
*Streptococcus agalactiae*	Lipoprotein signaling peptidase	−8.6 *	−5.8	−5.7
	Catabolite control protein	−8.1 *	−6.0	−5.7
	Large ribosomal subunit protein L19	−7.1 *	−4.8	−5.3
	Phosphopentomutase	−6.5 *	−5.6	−5.8
*Vibrio anguillarum*	Dihydrofolate reductase	−9.0 *	−4.6	−5.9
*Staphylococcus xylosus*	Glutamine synthetase	−10.3 *	−6.6 *	−6.4

* Bold type and asterisks indicate values that we consider low enough to indicate stable bonds.

**Table 2:** Antimicrobial activity of cell-free supernatants of probiotic strains against fish pathogens.

Strain	Dilution		*Pseudomonas aeruginosa*	*Aeromonas veronii*
Control	-	OD600	0.356 ± 0.036	0.733 ± 0.072
		%	100%	100%
		%	97%	102%
MT55	1/2	OD600	0	0
		%	0% *	0% *
	1/4	OD600	0	0.413 ± 0.029
		%	0% *	56% *
MT155	1/2	OD600	0	0
		%	0% *	0% *
	1/4	OD600	0	0.352 ± 0.042
		%	0% *	48% *

* *p* < 0.05.

## Data Availability

The original contributions presented in this study are included in this article. Further inquiries can be directed to the corresponding author.

## Supplementary Materials

The following supporting information can be downloaded at https://www.mdpi.com/article/10.3390/ijms26125811/s1.
