# Supplementary material for: Bacillibactin, a Potential Bacillus-Based Antibacterial Non-Ribosomal Peptide: In Silico Studies for Targeting Common Fish Pathogens
Source: Int J Mol Sci. 2025 Jun 17;26(12):5811. doi: 10.3390/ijms26125811 (PMC12192977; doi:10.3390/ijms26125811)
Supplement: Supplementary file 1 [file ijms-26-05811-s001.zip › ijms-3648151-supplementary.pdf]

Supplementary Table S1. Detailed description of productive poses for dihydrofolate reductase

**Dihydrofolate reductase and bacillibactin interactions**			
Protein and ligand atoms	d (Å)	Interaction type	θ (°)
A:**ARG53***:NH2 - A:Bacillibactin:O1	4,09922	Attractive Charge	
A:Bacillibactin:H37 - A:PRO26:O	2,28873	Conventional Hydrogen Bond	157,094
A:Bacillibactin:H17 - A:ILE51:O	2,43749	Conventional Hydrogen Bond	135,33
A:Bacillibactin:O11 - A:MET21:SD	3,68808	Conventional Hydrogen Bond	
A:Bacillibactin:O11 - A:SER50:OG	3,01863	Conventional Hydrogen Bond	
A:ARG53:HE - A:Bacillibactin:O4	2,01048	Conventional Hydrogen Bond	132,036
A:ARG53:HH21 - A:Bacillibactin:O4	2,44977	Conventional Hydrogen Bond	126,715
A:Bacillibactin:C17 - A:SER50:O	3,5085	Carbon Hydrogen Bond	
A:HIS24:CD2 - A:Bacillibactin:O12	3,55592	Carbon Hydrogen Bond	
A:ALA30:CB - A:Bacillibactin	3,79702	Pi-Sigma	
A:Bacillibactin - A:PHE32	5,00663	Pi-Pi T-shaped	
A:Bacillibactin - A:PHE29	5,00262	Pi-Pi T-shaped	
A:Bacillibactin - A:ILE51	4,40103	Pi-Alkyl	
**Dihydrofolate reductase and fengycin interactions**			
Protein and ligand atoms	d (Å)	Interaction type	θ (°)
:Fengycin:H23 - A:VAL74:O	2,94805	Conventional Hydrogen Bond	105,298
:Fengycin:H65 - A:ILE76:O	2,37297	Conventional Hydrogen Bond	120,868
:Fengycin:H5 - A:LEU55:O	3,05222	Conventional Hydrogen Bond	123,62
:Fengycin:H5 - A:**ARG58***:O	2,32143	Conventional Hydrogen Bond	107,434
A:GLY73:HN - :Fengycin:O5	2,32947	Conventional Hydrogen Bond	121,814
:Fengycin - A:PRO54	4,24245	Alkyl	
A:PRO54 - :Fengycin	4,57118	Alkyl	
A:PRO54 - :Fengycin	4,91463	Alkyl	
A:PRO56 - :Fengycin	4,10119	Alkyl	
**Dihydrofolate reductase and surfactin interactions**			
Protein and ligand atoms	d (Å)	Interaction type	θ (°)
:Surfactin:H33 - A:**GLU82***:OE1	2,24428	Conventional Hydrogen Bond	136,035
:Surfactin:H37 - A:SER78:OG	2,01499	Conventional Hydrogen Bond	165,712
:Surfactin:H41 - A:ILE76:O	2,20627	Conventional Hydrogen Bond	150,232

The table uses color coding to indicate the quality of molecular interactions based on geometric criteria: green indicates high-quality interactions with a distance (d) ≤ 3.0 Å and an angle (θ) ≥ 120°, representing strong hydrogen bonds or contacts. Yellow represents moderate interactions with distances between 3.1–3.4 Å and angles ranging from 90–119°. Red denotes weak or non-productive interactions, where the distance exceeds 3.4 Å or the angle is less than 90°. The important residues in the catalytic sites are presented in bold and red text along with an asterisk (*).

Supplementary Table S2. Detailed description of productive poses for proaerolysin

**Proaerolysin and bacillibactin interactions**			
Protein and ligand atoms	d (Å)	Interaction type	θ (°)
A:ARG397:NH2 - A:Bacillibactin:O2	5,49767	Attractive Charge	
A:ASN178:HD22 - A:Bacillibactin:O10	2,66151	Conventional Hydrogen Bond	92,34
A:GLY346:HN - A:Bacillibactin:O15	2,37148	Conventional Hydrogen Bond	148,193
A:TYR348:HN - A:Bacillibactin:O14	3,02314	Conventional Hydrogen Bond	91,991
A:**ARG356***:HE - A:Bacillibactin:O12	2,38511	Conventional Hydrogen Bond	156,575
A:**ARG356***:HH21 - A:Bacillibactin:O12	2,46878	Conventional Hydrogen Bond	148,098
A:Bacillibactin:H16 - A:ASP311:OD2	2,08333	Conventional Hydrogen Bond	141,444
A:Bacillibactin:H37 - A:ASP182:OD2	3,00852	Conventional Hydrogen Bond	134,058
A:Bacillibactin:H38 - A:TYR348:O	2,16741	Conventional Hydrogen Bond	129,955
A:Bacillibactin:O9 - A:LEU393:O	3,19955	Conventional Hydrogen Bond	
A:Bacillibactin:H27 - A:GLY346:O	2,89208	Conventional Hydrogen Bond	155,266
A:Bacillibactin:H39 - A:VAL344:O	2,91837	Conventional Hydrogen Bond	91,759
A:Bacillibactin - A:PRO181	4,36653	Pi-Alkyl	
A:Bacillibactin - A:LYS242	5,43275	Pi-Alkyl	
A:Bacillibactin - A:**PRO347***	4,70885	Pi-Alkyl	
A:Bacillibactin - A:**ARG356***	4,49097	Pi-Alkyl	
**Proaerolysin and fengycin interactions**			
Protein and ligand atoms	d (Å)	Interaction type	θ (°)
:Fengycin:H51 - A:VAL312:O	2,37788	Conventional Hydrogen Bond	93,822
:Fengycin:H98 - A:GLU84:OE1	2,34434	Conventional Hydrogen Bond	119,323
:Fengycin:H97 - A:ASP7:OD1	2,9468	Conventional Hydrogen Bond	111,676
:Fengycin:H57 - A:ASP311:OD2	2,34666	Conventional Hydrogen Bond	136,11
A:THR87:HG1 - :Fengycin:O10	2,88798	Conventional Hydrogen Bond	98,938
A:**ARG356***:HH21 - :Fengycin:O14	2,68007	Conventional Hydrogen Bond	131,669
A:ARG397:HE - :Fengycin:O5	2,53663	Conventional Hydrogen Bond	99,422
A:ARG397:HH22 - :Fengycin:O5	2,71784	Conventional Hydrogen Bond	90,656
A:ASP7:OD2 - :Fengycin	4,53953	Pi-Anion	

:Fengycin - A:LEU393	5,23087	Alkyl	
A:**ARG356*** - :Fengycin	4,09397	Alkyl	
A:**ARG356*** - :Fengycin	4,48972	Alkyl	
A:**ARG356*** - :Fengycin	5,42834	Alkyl	
:Fengycin - A:PRO395	5,00244	Pi-Alkyl	
A:TYR348 - :Fengycin	5,1598	Pi-Alkyl	
A:**TYR357*** - :Fengycin	5,14612	Pi-Alkyl	
**Proaerolysin and surfactin interactions**			
Protein and ligand atoms	d (Å)	Interaction type	θ (°)
:Surfactin:H93 - A:GLY346:O	2,5176	Conventional Hydrogen Bond	97
A:SER354:HG - :Surfactin:O12	2,14951	Conventional Hydrogen Bond	120,866
A:**ARG356***:HN - :Surfactin:O12	2,671	Conventional Hydrogen Bond	148,357
:Surfactin:C50 - A:TYR348	3,86071	Pi-Sigma	
:Surfactin:C3 - A:LYS242	4,74352	Alkyl	
:Surfactin:C3 - A:**PRO347***	4,18942	Alkyl	
A:**PRO347*** - :Surfactin	4,59654	Alkyl	
A:**TYR348*** - :Surfactin	4,87936	Pi-Alkyl	

The table uses color coding to indicate the quality of molecular interactions based on geometric criteria: green indicates high-quality interactions with a distance (d) ≤ 3.0 Å and an angle (θ) ≥ 120°, representing strong hydrogen bonds or contacts. Yellow represents moderate interactions with distances between 3.1–3.4 Å and angles ranging from 90–119°. Red denotes weak or non-productive interactions, where the distance exceeds 3.4 Å or the angle is less than 90°. The important residues in the catalytic sites are presented in bold and red text along with an asterisk (*).

Supplementary Table S3. Detailed description of productive poses for glutamine synthetase

**Glutamine synthetase and bacillibactin interactions**			
Protein and ligand atoms	d (Å)	Interaction type	θ (°)
A:Bacillibactin:N2 - A:GLU186:OE2	4,76128	Attractive Charge	
A:Bacillibactin:N5 - A:GLU186:OE2	3,93502	Attractive Charge	
A:Bacillibactin:N3 - A:GLU134:OE1	4,83372	Attractive Charge	
A:Bacillibactin:N3 - A:GLU198:OE2	5,08767	Attractive Charge	
A:Bacillibactin:N6 - A:GLU134:OE1	4,06118	Attractive Charge	
A:Bacillibactin:N6 - A:GLU191:OE2	5,4877	Attractive Charge	
A:Bacillibactin:N6 - A:GLU198:OE2	4,06575	Attractive Charge	
A:HIS189:NE2 - A:Bacillibactin:O2	5,17952	Attractive Charge	
A:Bacillibactin:H37 - A:SER188:O	2,61397	Conventional Hydrogen Bond	110,631

A:Bacillibactin:H39 - A:GLU306:OE2	2,70286	Conventional Hydrogen Bond	94,892
A:TYR158:HH - A:Bacillibactin:O9	2,7516	Conventional Hydrogen Bond	113,124
A:ARG318:HE - A:Bacillibactin:O12	2,44029	Conventional Hydrogen Bond	122,054
A:ARG318:HE - A:Bacillibactin:O15	2,3076	Conventional Hydrogen Bond	122,548
A:ARG318:HH21 - A:Bacillibactin:O12	2,6125	Conventional Hydrogen Bond	118,831
A:**ARG323***:HH21 - A:Bacillibactin:O12	2,74428	Conventional Hydrogen Bond	101,589
A:SER327:HG - A:Bacillibactin:O8	2,28958	Conventional Hydrogen Bond	146,958
A:ARG337:HH22 - A:Bacillibactin:O12	2,35468	Conventional Hydrogen Bond	139,261
A:Bacillibactin:C18 - A:GLU134:OE1	3,69833	Carbon Hydrogen Bond	
A:GLU134:OE1 - A:Bacillibactin	4,38967	Pi-Anion	
A:GLU186:OE1 - A:Bacillibactin	3,97926	Pi-Anion	
A:GLU191:OE2 - A:Bacillibactin	3,8453	Pi-Anion	
A:Bacillibactin - A:PHE159	4,25546	Pi-Pi Stacked	
A:**HIS247*** - A:Bacillibactin	4,38494	Pi-Pi T-shaped	
**Glutamine synthetase and fengycin interactions**			
Protein and ligand atoms	d (Å)	Interaction type	θ (°)
A:ARG93:HH21 - :Fengycin:O2	2,28834	Conventional Hydrogen Bond	134,112
A:LYS115:HZ2 - :Fengycin:O8	2,44883	Conventional Hydrogen Bond	120,993
A:LYS122:HZ3 - :Fengycin:O17	2,97491	Conventional Hydrogen Bond	109,283
A:THR208:HG1 - :Fengycin:O16	3,04182	Conventional Hydrogen Bond	121,41
:Fengycin:H92 - A:GLU128:OE1	1,94202	Conventional Hydrogen Bond	160,004
:Fengycin:H88 - A:GLU128:OE1	2,28893	Conventional Hydrogen Bond	137,898
:Fengycin:H65 - A:ASP75:OD1	2,57459	Conventional Hydrogen Bond	152,786
:Fengycin:H97 - A:GLY255:O	2,26839	Conventional Hydrogen Bond	147,784
:Fengycin:H57 - A:TRP79:O	2,12197	Conventional Hydrogen Bond	157,151
A:THR78:CA - :Fengycin:O3	3,37451	Carbon Hydrogen Bond	
:Fengycin:C39 - A:SER127:O	3,46226	Carbon Hydrogen Bond	
A:**LYS202*** - :Fengycin	5,23707	Alkyl	
A:ALA204 - :Fengycin	4,84265	Alkyl	
A:ALA204 - :Fengycin	4,19665	Alkyl	
**Glutamine synthetase and surfactin interactions**			
Protein and ligand atoms	d (Å)	Interaction type	θ (°)

A:**LYS202***:HN - :Surfactin:O13	2,74015	Conventional Hydrogen Bond	102,804
:Surfactin:H93 - A:**LYS202***:O	2,80818	Conventional Hydrogen Bond	91,276
A:ALA204:CA - :Surfactin:O3	3,40121	Carbon Hydrogen Bond	
:Surfactin:C36 - A:PHE6	3,86594	Pi-Sigma	
A:VAL80 - :Surfactin	5,45168	Alkyl	
A:PHE6 - :Surfactin	4,66015	Pi-Alkyl	
A:PHE183 - :Surfactin:C3	5,01193	Pi-Alkyl	
A:TYR203 - :Surfactin	4,89316	Pi-Alkyl	
A:TYR203 - :Surfactin:C50	5,27934	Pi-Alkyl	

The table uses color coding to indicate the quality of molecular interactions based on geometric criteria: green indicates high-quality interactions with a distance (d) ≤ 3.0 Å and an angle (θ) ≥ 120°, representing strong hydrogen bonds or contacts. Yellow represents moderate interactions with distances between 3.1–3.4 Å and angles ranging from 90–119°. Red denotes weak or non-productive interactions, where the distance exceeds 3.4 Å or the angle is less than 90°. The important residues in the catalytic sites are presented in bold and red text along with an asterisk (*).

Supplementary Table S4. Grid-box dimensions and axes

	Bacillibactin	Fengycin	Surfactin
Catabolite control protein	center_x = -5.665	center_x = -5.665	center_x = -5.665
	center_y = 15.065	center_y = 15.065	center_y = 15.065
	center_z = 88.667	center_z = 88.667	center_z = 88.667
	size_x = 40	size_x = 40	size_x = 40
	size_y = 40	size_y = 40	size_y = 40
	size_z = 40	size_z = 40	size_z = 40
D-alanine-D-alanine ligase	center_x = -24.884	center_x = -24.884	center_x = -24.884
	center_y = 6.728	center_y = 6.728	center_y = 6.728
	center_z = -2.148	center_z = -2.148	center_z = -2.148
	size_x = 40	size_x = 40	size_x = 40
	size_y = 40	size_y = 40	size_y = 40
	size_z = 40	size_z = 40	size_z = 40
Dihydrofolate reductase	center_x = 48.926	center_x = 48.926	center_x = 48.926
	center_y = -4.553	center_y = -4.553	center_y = -4.553
	center_z = -32.494	center_z = -32.494	center_z = -32.494
	size_x = 40	size_x = 40	size_x = 40
	size_y = 40	size_y = 40	size_y = 40
	size_z = 40	size_z = 40	size_z = 40

Glutamine synthetase	center_x = -10.091	center_x = -10.091	center_x = -10.091
	center_y = 53.286	center_y = 53.286	center_y = 53.286
	center_z = 56.443	center_z = 56.443	center_z = 56.443
	size_x = 54	size_x = 54	size_x = 54
	size_y = 42	size_y = 42	size_y = 42
	size_z = 40	size_z = 40	size_z = 40
Large ribosomal subunit protein L19	center_x = 105.926	center_x = 105.926	center_x = 105.926
	center_y = 164.857	center_y = 164.857	center_y = 164.857
	center_z = 211.137	center_z = 211.137	center_z = 211.137
	size_x = 42	size_x = 42	size_x = 42
	size_y = 40	size_y = 40	size_y = 40
	size_z = 40	size_z = 40	size_z = 40
Lipoprotein signaling peptidase	center_x = 1.254	center_x = 1.254	center_x = 1.254
	center_y = -2.584	center_y = -2.584	center_y = -2.584
	center_z = 6.769	center_z = 6.769	center_z = 6.769
	size_x = 64	size_x = 64	size_x = 64
	size_y = 64	size_y = 64	size_y = 64
	size_z = 72	size_z = 72	size_z = 72
Phosphopentomutase	center_x = 8.374	center_x = 8.374	center_x = 8.374
	center_y = 42.234	center_y = 42.234	center_y = 42.234
	center_z = 3.595	center_z = 3.595	center_z = 3.595
	size_x = 40	size_x = 40	size_x = 40
	size_y = 40	size_y = 40	size_y = 40
	size_z = 40	size_z = 40	size_z = 40
Proaerolysin	center_x = 0.59	center_x = 0.59	center_x = 0.59
	center_y = 59.946	center_y = 59.946	center_y = 59.946
	center_z = 28.894	center_z = 28.894	center_z = 28.894
	size_x = 40	size_x = 40	size_x = 40
	size_y = 52	size_y = 52	size_y = 52
	size_z = 40	size_z = 40	size_z = 40
S-adenosylmethionine synthase	center_x = 8.808	center_x = 8.808	center_x = 8.808
	center_y = 5.413	center_y = 5.413	center_y = 5.413

	center_z = 5.413	center_z = 5.413	center_z = 5.413
	size_x = 46	size_x = 46	size_x = 46
	size_y = 40	size_y = 40	size_y = 40
	size_z = 44	size_z = 44	size_z = 44
